# Sialic acids Neu5Ac and KDN in adipose tissue samples from individuals following habitual vegetarian or non-vegetarian dietary patterns

**DOI:** 10.1038/s41598-023-38102-z

**Published:** 2023-08-03

**Authors:** Gerardo N. Guerrero-Flores, Fabio J. Pacheco, Danilo S. Boskovic, Sandaly O. S. Pacheco, Guangyu Zhang, Gary E. Fraser, Fayth L. Miles

**Affiliations:** 1 Interdisciplinary Center for Research in Health and Behavioral Sciences, School of Medicine, Universidad Adventista del Plata, 3103 Libertador San Martín, Entre Ríos Argentina; 2 Faculty of Medical Sciences, Universidad Nacional de Rosario (UNR), 3100 Rosario, Argentina; 3 Institute for Food Science and Nutrition, Universidad Adventista del Plata, 3103 Libertador San Martín, Entre Ríos Argentina; 4 Division of Biochemistry, Department of Basic Sciences, School of Medicine, Loma Linda University, Loma Linda, CA 92350 USA; 5 Center for Nutrition, Healthy Lifestyles and Disease Prevention, School of Public Health, Loma Linda University, Loma Linda, CA 92350 USA; 6 Adventist Health Study, Loma Linda University, Loma Linda, CA 92350 USA; 7 Department of Medicine, School of Medicine, Loma Linda University, Loma Linda, CA 92350 USA

**Keywords:** Glycobiology, Molecular medicine

## Abstract

Sialic acids (Sias) are a class of sugar molecules with a parent nine-carbon neuraminic acid, generally present at the ends of carbohydrate chains, either attached to cellular surfaces or as secreted glycoconjugates. Given their position and structural diversity, Sias modulate a wide variety of biological processes. However, little is known about the role of Sias in human adipose tissue, or their implications for health and disease, particularly among individuals following different dietary patterns. The goal of this study was to measure *N*-Acetylneuraminic acid (Neu5Ac), *N*-Glycolylneuraminic acid (Neu5Gc), and 2-keto-3-deoxy-d-glycero-d-galacto-nononic acid (KDN) concentrations in adipose tissue samples from participants in the Adventist Health Study-2 (AHS-2) and to compare the abundance of these Sias in individuals following habitual, long-term vegetarian or non-vegetarian dietary patterns. A method was successfully developed for the extraction and detection of Sias in adipose tissue. Sias levels were quantified in 52 vegans, 56 lacto-vegetarians, and 48 non-vegetarians using LC–MS/MS with Neu5Ac-D-1,2,3-^13^C_3_ as an internal standard. Dietary groups were compared using linear regression. Vegans and lacto-ovo-vegetarians had significantly higher concentrations of Neu5Ac relative to non-vegetarians. While KDN levels tended to be higher in vegans and lacto-ovo-vegetarians, these differences were not statistically significant. However, KDN levels were significantly inversely associated with body mass index. In contrast, Neu5Gc was not detected in human adipose samples. It is plausible that different Neu5Ac concentrations in adipose tissues of vegetarians, compared to those of non-vegetarians, reflect a difference in the baseline inflammatory status between the two groups. Epidemiologic studies examining levels of Sias in human adipose tissue and other biospecimens will help to further explore their roles in development and progression of inflammatory conditions and chronic diseases.

## Introduction

The sialic acids (Sias) are a diverse family of sugar molecules derived from the parent nine-carbon compound, neuraminic acid^1–3^. Given their position and structural diversity, Sias modulate a wide variety of biological processes relevant to health and disease, and consequently are of growing interest in the field of glycobiology^4–6^.

*N*-Acetylneuraminic acid (Neu5Ac) and its hydroxylated derivative *N*-glycolylneuraminic acid (Neu5Gc) are the most prevalent Sia forms in mammals^7^. Spontaneously occurring derivatives of Sia are formed by modification of the parent Sia with phosphate, sulphate, lactyl, or acetyl groups^4,8,9^. Humans, however, do not produce Neu5Gc, because of an absence of cytidine monophosphate-*N*-acetylneuraminic acid hydroxylase which converts CMP-Neu5Ac to CMP-Neu5Gc^3,10^. Nevertheless, Neu5Gc can be metabolically incorporated into human tissues from dietary sources, mainly red meats, and dairy products^11–13^. The more recently described Sia family member, 2-keto-3-deoxy-d-glycero-d-galacto-nononic acid (KDN), is generally expressed in mammals at low levels.

The negatively charged Sias are involved in interactions with Sia-binding proteins in multiple biological processes including immunity and cancer^14^. Moreover, the negative surface charges of Sias can mask glycan ligands or serve as attachment sites for pathogens and toxins^4,15,16^. In addition to their presence in various mammalian cells and tissues, Sias may be present in various body fluids including saliva, urine, plasma, milk, and cerebrospinal fluid^17–20^.

The production and abundance of Sias appears to be site-specific and context dependent^8,21^. Higher Sia levels may be due either to increased synthesis and dietary intake or due to upregulated sialidase function (releasing free Sia). On the one hand, dietary supplementation in animals was shown to be protective against atherosclerotic and cardiovascular disease (CVD), and associated with lower inflammatory tone^22–24^. This might suggest that higher levels of Sias have a moderating effect on inflammation. However, Sias are elevated in the serum of patients with coronary artery disease and may correlate with disease severity^25^. Additionally, increasing levels of Sias have been associated with inflammation, hemolytic-uremic syndrome, diabetes^26–28^, CVD^29–31^, and cancer^6,32,33^. In part, this is consistent with a recognized association between elevated sialidase function and inflammation^34–38^. In this context, Sias may serve as general indicators of an inflammatory acute-phase response^39^.

In contrast to serum, limited information is available about Sias in human adipose tissues. In addition to its function as the primary site for excess energy storage, adipose tissue also serves as an endocrine organ synthesizing several biologically active agents involved in the regulation of metabolic homeostasis^40,41^. The cellular constituents of adipose tissue include adipocytes, blood cells, endothelial cells, pericytes, and adipose precursor cells^40,42–44^. Excessive adipose accumulation can lead to abnormal secretion of adipocytokines or other signaling molecules, which may trigger processes associated with diabetes, cancer, and cardiovascular diseases (CVDs)^40–42,45,46^. Adiposity is the basis for increased body mass index (BMI), which is known to be a major risk factor for CVD, diabetes, cancer and other diseases^47,48^. At present, the functional roles of Sias are unclear in the context of obesity, chronic inflammation, and CVD.

While it is not clear how human dietary patterns impact Sias levels, it seems reasonable to expect that Sias levels are regulated either by components of the diet or by diet-induced changes in adiposity and inflammatory responses. Consumption of red and processed meats, and in some cases dairy, has been associated with heart disease, diabetes, and cancer (particularly colorectal cancer)^49,49–52^. Vegetarians have more favorable cardiometabolic profiles and lower risks for type 2 diabetes, CVD, and various types of cancers including colorectal, gastrointestinal, breast, prostate, lung and female-specific cancers^49,50,53,54^. From this perspective, differences in Sias between dietary groups may help to explain the more favorable health outcomes associated with a vegetarian dietary pattern. The extraction and quantification of Sias from human adipose samples, particularly, may shed light on the role of Sias in metabolic health and the development of chronic diseases.

The goal of this study was to determine Neu5Ac, Neu5Gc, and KDN concentrations in adipose tissue samples from participants of the Adventist Health Study-2 (AHS-2) and to compare the abundance of these Sias in 156 individuals who follow habitual, long-term vegetarian or non-vegetarian dietary patterns. In order to accomplish this, a method for measurement of Sias was developed and optimized using LC–MS/MS.

## Materials and methods

### Materials

The Accucore HILIC column (150 mm × 4.6 mm, 2.6 μm particles) was obtained from Thermo Scientific (Waltham, MA) and connected with an Accucore HILIC precolumn (10 mm × 4.6 mm, 2.6 μm). Ammonium formate, trifluoroacetic acid (TFA), and acetonitrile were obtained from Fisher Scientific (Carlbad, CA). Neu5Ac-D-1,2,3-^13^C_3_, KDN, Neu5Ac, Neu5Gc, and methanol were purchased from Sigma-Aldrich (St. Louis, MI), and chloroform was obtained from EMD Millipore (Burlington, MA). Milli-Q water (GenPure Pro ultrapure water system with UV-photo-oxidation and TOC monitor, by Thermo Scientific Inc) was used throughout the study protocol.

### Ethical approvals

All procedures associated with this project were conducted following the international ethical standards proposed by the Helsinki protocol for human research and informed consent was obtained from all participants. This study was reviewed and approved by the Institutional Review Board of Loma Linda University School of Medicine (IRB #5190039).

### Adipose tissue samples

The present study measured Sias concentrations in 156 human adipose tissue samples from participants of the Adventist Health Study-2 (AHS-2) cohort. The AHS-2 cohort was established between 2002 and 2007 and consists of over 96,000 Seventh Day Adventists in the USA and Canada, with a considerable proportion (~ 50%) of vegetarians. Study participants completed a food frequency questionnaire at baseline and were defined as vegan (n = 52; strict vegetarians with complete avoidance of all animal products or consumption < 1/month), lacto-ovo-vegetarians (n = 56; avoidance of all flesh meats but consuming dairy and/or eggs ≥ 1/month) and non-vegetarians (n = 48; consuming flesh meats, not only fish,  ≥ 1/month). Sociodemographic information and body mass index (BMI) were calculated from the baseline questionnaire. The current study included selection of approximately equal numbers of samples from vegan, lacto-ovo-vegetarian, and non-vegetarian participants who previously provided biospecimens, including adipose tissue and blood, in one of two sub-studies. The first was the Biological Manifestations of Religion and Health study, where participants were recruited to clinic sites in Southern California^55^. The second sub-study included pilot work with biospecimen collections from field clinics and church halls^56^. Adipose tissue samples were obtained by aspirating a subcutaneous sample of fat from the buttock with a needle and syringe, and were maintained frozen in liquid nitrogen^57^. Samples of adipose tissue from cow, pork, lamb, and poultry were obtained from local supermarkets in Southern California, USA.

### Preparation of standard samples

Sample standards were prepared by dilution in Milli-Q water. Neu5Ac-D-1,2,3-^13^C_3_ was used as an internal standard (IS), due to its similar structure and behavior to analytes during chromatography separation of Sias, thereby enabling corrections for possible ion suppression/enhancement effects^58,59^. Moreover, the internal standard provides internal quality control and facilitates correction for unaccounted measurement variables. Additionally, positive controls with IS (30 ng/mL) in water were run before, during, and after each batch analysis.

### Standard calibration curves

Calibration curves with varying Sia concentrations were prepared. To improve the quantification range of the LC–MS/MS equipment, two separate calibration curves with good linearity in the low and high ranges were constructed for Neu5Ac and KDN. Additionally, a fixed concentration of IS was added to each calibrator. The Neu5Gc standard concentrations were: 0.0004, 0.0005, 0.00078, 0.0010, 0.0015, 0.0021, 0.0029, 0.0042, 0.0058, 0.0081, 0.0114, and 0.016 μg/mL. The KDN standard concentrations were: 0.0004, 0.0005, 0.0010, 0.0015, 0.0021, 0.0029, 0.0042, 0.0058, 0.0081, 0.0114, and 0.0160 μg/mL for low range, and 0.0050, 0.0100, 0.0150, 0.0200, 0.0400, 0.0600, 0.0900, 0.0120, 0.0160, 0.20, and 0.26 μg/mL for high range. Similarly, the Neu5Ac standard concentrations were: 0.001, 0.0019, 0.0036, 0.013, 0.091, 0.174, 0.330, 0.629, and 1.2 μg/mL for low range, and 0.8, 1.6, 2.4, 3.2, 4.0, 4.8, 5.6, 6.4, and 7.2 μg/mL for high range. After correction for IS peak amplitude variations, the LC–MS/MS peak signal amplitudes for KDN, Neu5Ac, or Neu5Gc were used to construct the calibration lines. In addition, IS was injected as a reference at regular intervals^60^ for added quality control. Supplementary Fig. S4 presents the LC–MS/MS calibration curves, with detector response as a function of the concentrations of Neu5Ac, KDN, and Neu5Gc. The calculated lower limits of detection were ≥ 2.0 ng/mL for all three Sias^61^. The lower ranges of standard lines were extended by employing ln-ln transformations. The correlation coefficients (*r*^2^) for all three Sias were > 0.980.

### Extraction of Neu5Ac, Neu5Gc, and KDN from adipose tissue

**Figure 1:**
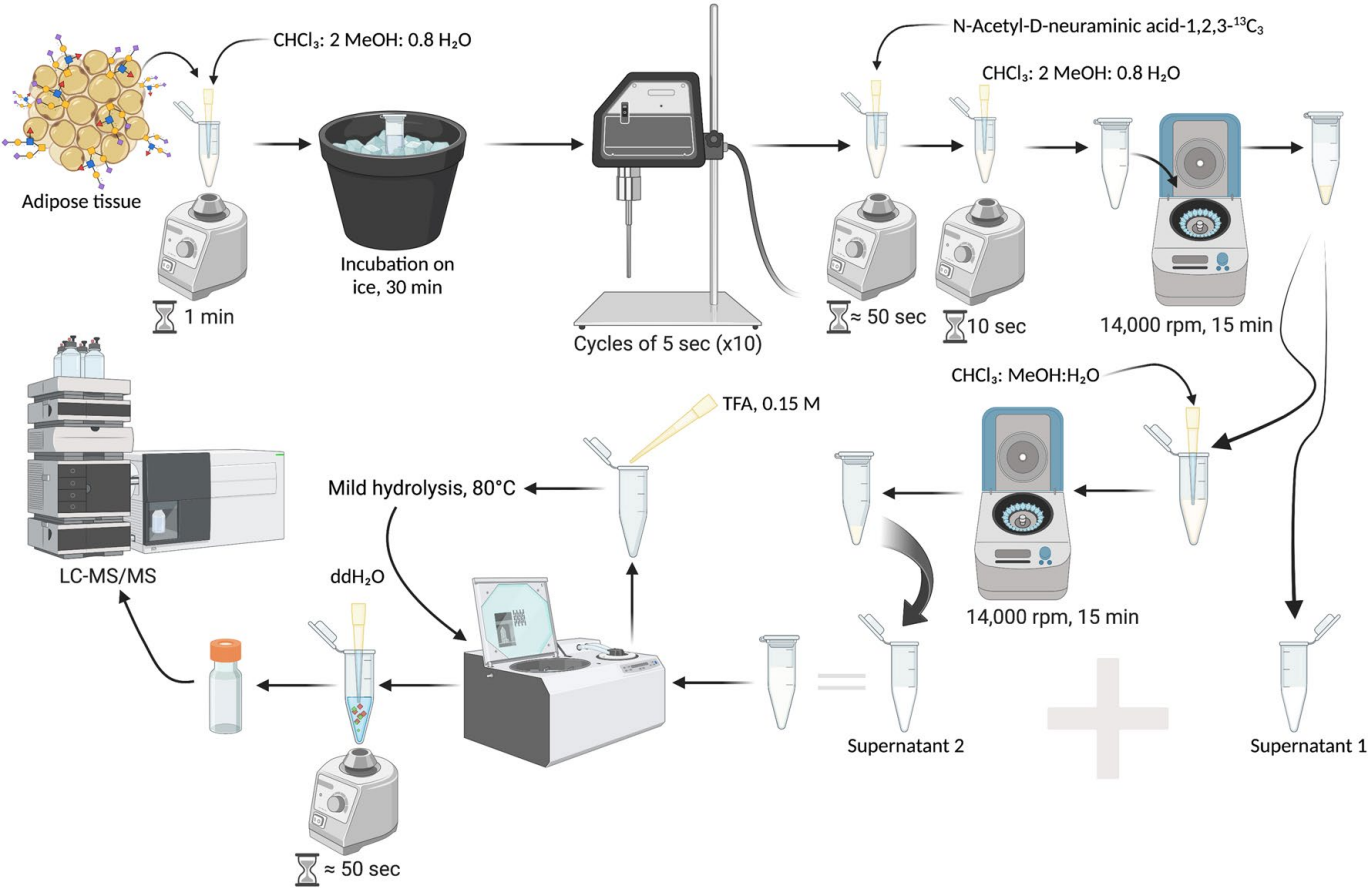
Processing of samples for the extraction, detection, and quantification of Sias from adipose tissue using LC/MS/MS. Created with BioRender.com.

Sample processing is illustrated in Fig. 1. A solution of 100 μL of chloroform, methanol, and water (1:2:0.8) was added to human or animal samples and vortexed thoroughly for 1 min, followed by incubation for 30 min on ice before sonication. Ten sonication cycles of 5 s each with an amplitude of 20% were performed (120 Sonic Dismembrator, Fisher Scientific Sonicator) producing a mixture of free and conjugated Sias. After sonication, 20 μL (150 ng/mL) of the internal standard was added to a final concentration of 30 ng/mL, to help control for extraction, loading, and detection variables^62^. Then, the sample was vortexed for ~ 50 s. At this point, a solution of chloroform in water (26.4 μL) was added to generate a final concentration of 2:2:1.8 for chloroform, methanol, and water, respectively. Then, the sample was vortexed for 10 s and centrifuged at 14,000 rpm for 15 min. The first supernatant was separated (70 μL) and 100 μL of chloroform, methanol, and water (1:2:0.8) was added to the remaining volume. The second round of centrifugation was followed by supernatant separation (50 μL). The supernatant fractions were combined and dried by SpeedVac (Savant SC 110A, Thermo Electron Corporation). After drying, TFA 0.15 M (100 μL) was added to the sample and incubated at 80 °C (Labnet, Vortemp 56 Shaking incubator) with shaking at 400 rpm for 2 h for release of Sias from lipids or proteins by hydrolysis^27^. The sample was spun down and evaporated using SpeedVac. Then this dried processed sample was reconstituted in 100 μL of water and analyzed by LC–MS/MS.

### LC–MS/MS quantification of sialic acids

Measurement of Sias was done using LC–MS/MS, following FDA Bioanalytical Method Guidelines^63–66^. The LC–MS/MS based approach was chosen with some adaptations from previous methods based on its selectivity, sensitivity, and reproducibility in identifying Sias^27,67–69^. Briefly, the instrumentation included an Agilent 1200 HPLC coupled with a triple quadruple mass spectrometer 6410 Agilent Technologies detector (Santa Clara, CA). Data processing was performed using Agilent Mass Hunter Software, version B.07.01.

The column temperature was set at 30 °C using an Accucore HILIC column (150 mm × 4.6 mm, 2.6 μm particles) connected with an Accucore HILIC precolumn (10 mm × 4.6 mm, 2.6 μm). The mobile phase flow rate was set to 0.3 mL/min. The mobile phase consisted of solution A (10% ammonium formate in water), and solution B (10% of ammonium formate in acetonitrile) with the following protocol: during the first min, 2% B; 1–2 min, gradient 2–90% B; 2–4 min, 90% B; 4–5 min, gradient 90–2% B; 5–7 min, 2% B. (See Supplementary Table S1, and Fig. S5). A blank with water was run as a negative control between each individual standard or sample run. The injection volume for standards and samples was set at 20 μL.

Chromatography and electrospray ion source (ESI) parameters were used as previously described^20,27^. Quantification of Sias from adipose tissue, including that of the added internal standard (Neu5Ac-D-1,2,3-^13^C_3_) (IS), was carried out in negative electrospray ionization (ESI) mode. (Supplementary Fig. S3). The ion spray voltage was operated at − 4 kV, and the source temperature was 300 °C. The nebulizer was set at 35 psi and the gas flow setting was 11 L/min. The singly charged [M-H]- parent ions were identified for: KDN at *m/z* 267.1, Neu5Ac at *m/z* 308.0, Neu5Gc at *m/z* 324.1, and Neu5Ac-D-1,2,3-^13^C_3_ at *m/z* 311.1. Selected daughter ions for KDN, Neu5Ac, Neu5Gc, and Neu5Ac-D-1,2,3-^13^C_3_ were at *m/z* 87.1, 87.0, 116.1, and 90.0, respectively. (Supplementary Table S2). Multiple reaction monitoring (MRM) mode was used. Peak areas for KDN, Neu5Ac, or Neu5Gc were corrected for variations in peak areas of the IS (Neu5Ac-D-1,2,3-^13^C_3_), followed by calculations of respective Sia concentrations based on the regression lines of standards.

### Sialic acid levels in adipose tissue

Sias concentrations in samples were determined using standard calibration lines (Supplementary Fig. S4). The weight of human samples ranged from 5 to 30 mg, and animal samples ranged 10–20 mg, measured using an analytical balance (Mettler-Toledo, AB54-S). Sias concentrations were expressed in relative terms to the weight of the adipose tissue sample before extraction in (ng Sia)/(mg adipose) or ppm.

The standard lines were developed using varying respective Sias concentrations in the presence of a fixed concentration of 30 ng/ml for the internal standard (IS). Linearization was obtained following the natural logarithmic transformations of the MS detector signal intensity ratios $\left(\frac{S(Sia)}{S(IS)}\right)$ and known concentration ratios $\left(\frac{[Sia]}{[IS]}\right)$, to produce the line Eq. (1) in the standard format $y=mx+b$. Then a rearrangement, illustrated by Eq. (2), was employed to convert MS detector signal intensities for sample Sias with known IS to appropriate Sias concentrations (3), after taking dilution factor (DF) into account. The specific equations of the form (2) for each Sia are presented with Supplementary Fig. S4. Two different standard lines are employed for both Neu5Ac and for KDN measurements, to accommodate potential low and high concentration ranges.

1 $$ln\left(\frac{S(Sia)}{S(IS)}\right)=m*\mathit{ln}\left(\frac{\left[Sia\right]}{\left[IS\right]}\right)+b$$

2 $$\mathit{ln}\left(\frac{\left[Sia\right]}{\left[IS\right]}\right)=\left(\frac{1}{m}\right)*ln\left(\frac{S\left(Sia\right)}{S\left(IS\right)}\right)-\left(\frac{b}{m}\right)$$

3 $$\left[Sia\right]=DF*\left[IS\right]*{e}^{ln\left(\frac{\left[Sia\right]}{[IS]}\right)}$$

### Statistical analysis

Descriptive analyses were carried out adjusting for demographic data, comparing the three dietary groups—vegans, lacto-ovo-vegetarians, and non-vegetarians. To compare Sias between diet groups, sialic acids were first log-transformed, and then a linear regression model was fitted to examine the association between vegetarian dietary status (vegan or lacto-ovo-vegetarian vs non-vegetarian, and vegan vs lacto-ovo-vegetarian) and Sia abundance, where the Sia was the dependent/response variable, and dietary pattern was the predictor variable of interest. Covariates included race (Black vs non-Black), age at blood collection (continuous), gender (male vs female), batch (continuous), sub-study (Adventist Religion and Health vs pilot), and BMI (kg/m^2^; continuous). Beta coefficients and 95% confidence intervals were obtained for each diet group/Sia comparison, along with the adjusted predicted means (R package emmeans) and 95% confidence intervals, providing a marginal estimate of the log of the mean of untransformed Sia values for vegetarians and non-vegetarians, and subsequently back-transformed. Linear regression analysis was also used to examine associations of BMI with Sias, adjusting for race, age at blood collection, gender, batch, and sub-study. All analyses were conducted in R version 4.0.2.

## Results

### Separation of sialic acids

The chemical structures of the studied Sias are shown in Supplementary Fig. S1. The HPLC peaks for Neu5Ac, Neu5Gc, and KDN standards are well resolved using LC–MS/MS with stable retention times (Supplementary Fig. S2). The retention times for KDN, Neu5Gc, Neu5Ac-D-1,2,3-^13^C_3_, and Neu5Ac were found to be 5.028, 5.036, 5.070, and 5.079 min, respectively. The ESI mass spectra of parent and daughter ions, as shown in Supplementary Fig. S2, for Neu5Ac, KDN, Neu5Ac-D-1,2,3-^13^C_3_ and Neu5Gc were chosen because of their relative abundances^27,70–72^.

### Sialic acids from animal adipose tissue

Sias were extracted from adipose tissue samples from pig, lamb, cow, or chicken. All three Sias were released from adipose tissues and were measurable by the LC–MS/MS method (Supplementary Table S1). Total adipose tissue Sias (μg/mg) were highest in pork (5.669 μg/mg), followed by beef (4.958 μg/mg), lamb (3.592 μg/mg) and chicken (2.438 μg/mg). Neu5Ac was the major Sia form in all animal samples. As expected, Neu5Gc was not detected in chicken samples. (Supplementary Table S3).

### Study population characteristics

Characteristics of the study population are presented in Table 1. The study cohort included 156 participants distributed among vegan, lacto-ovo vegetarian, and non-vegetarian dietary groups. Some statistical differences were evident among dietary groups. A significantly lower proportion of Black participants were vegan (*p* = 0.0007). Compared to other dietary groups, the vegans had significantly lower mean BMI (*p* = 0.004) and significantly higher adipose Neu5Ac (*p* = 0.02) considering unadjusted means.

**Table 1:** Demographic characteristics of study population.

	Vegan	Lacto-ovo	Non-vegetarian	*p* value^1^
Participants	52 (33.3)	56 (35.8)	48 (30.7)	
Age (years)	61.3 (14.2)	58.1 (13.3)	60.8 (14.0)	0.45
Sex				
Female	35 (31.8)	40 (36.3)	35 (31.8)	
Male	17 (36.9)	16 (34.7)	13 (28.2)	0.81
Race				
Black	23 (22.7)	41 (40.5)	37 (36.6)	
Non-Black	29 (52.7)	15 (27.2)	11 (20.0)	0.0007
BMI (kg/m2)	27.5 (5.4)	31.2 (7.5)	31.6 (7.4)	0.004
KDN (ng/mg)	1.4 (0.91)	1.6 (1.5)	1.04 (1.06)	0.083
Neu5Ac (ng/mg)	40.2 (38.9)	36.5 (46.4)	20.6 (21.7)	0.024

Values are presented as n (%), or mean (SD).

^1^*p* value computed using chi-square test for categorical variables and ANOVA for continuous variables.

### Associations of sialic acids from human adipose tissue with dietary patterns and BMI

Neu5Ac and KDN were measured and quantified in adipose tissue samples of vegans, lacto-ovo-vegetarians, and non-vegetarians as described. These Sia concentrations (ng/mg) were compared between diet groups using linear regression analysis, and adjusted means were obtained (Table 2). Neu5Ac was significantly higher in adipose tissues from vegans (mean: 17.1, 95% CI 12.5–23.6) compared to non-vegetarians (mean: 9.8, 95% CI 7.1–13.7). Similarly, Neu5Ac was higher in adipose of lacto-ovo-vegetarians (mean: 12.7, 95% CI 9.2–17.5) compared to non-vegetarians (mean: 7.7, 95% CI 5.4–10.8) (Table 2). These means were statistically different (*p* = 0.01 and *p* = 0.02, respectively), even after inclusion of BMI as a covariate (Table 2). KDN concentrations were higher in vegans and lacto-ovo-vegetarians relative to non-vegetarians, though differences were not statistically significant. Moreover, linear regression revealed an inverse association between BMI and KDN (*p* = 0.02), independent of diet group (Table 3). Neu5Gc was not detected in any adipose samples of participants in any diet group.

**Table 2:** Adjusted means from linear regression model of associations of dietary patterns with Neu5Ac and KDN.

	Model 1^1^			Model 2^2^		
	Vegan	Non-vegetarian	*p* value	Vegan	Non-vegetarian	*p* value
Neu5Ac	17.13 (12.45, 23.58)	9.84 (7.09, 13.67)	0.01	17.55 (12.67, 24.30)	9.70 (6.96, 13.51)	0.01
KDN	0.79 (0.63, 0.99)	0.63 (0.50, 0.79)	0.15	0.78 (0.62, 0.98)	0.63 (0.50, 0.80)	0.19

	Vegan	Lacto-ovo	*p* value	Vegan	Lacto-ovo	*p* value
Neu5Ac	16.97 (12.08, 23.84)	15.20 (10.97, 21.06)	0.46	16.97 (12.08, 23.84)	16.44 (11.92, 22.69)	0.88
KDN	0.80 (0.62, 1.02)	0.82 (0.63, 1.07)	0.85	0.79 (0.61, 1.03)	0.85 (0.66, 1.09)	0.66

	Lacto-ovo	Non-vegetarian	*p* value	Lacto-ovo	Non-vegetarian	*p* value
Neu5Ac	12.71 (9.23, 17.49)	7.65 (5.40, 10.83)	0.02	12.55 (9.13, 17.24)	7.62 (5.39, 10.76)	0.02
KDN	0.74 (0.56, 0.97)	0.54 (0.40, 0.74)	0.1	0.73 (0.55, 0.95)	0.54 (0.40, 0.73)	0.11

Estimated marginal (least-squares) means (ng/mg) and 95% confidence intervals obtained from linear regression.

*BMI* body mass index.

^1^Adjusted for age at blood collection, sub-study, race, gender, and batch.

^2^Adjusted for covariates in model 1, in addition to BMI.

**Table 3:** Associations of BMI with KDN and Neu5Ac.

	Model 1^1^			Model 2^2^		
	β coefficient	Std. Err	*P* value	β coefficient	Std. Err	*p* value
KDN	− 0.023	0.009	0.013	− 0.020	0.009	0.02
Neu5Ac	− 0.020	0.01	0.09	− 0.016	0.012	0.18

Beta-coefficients obtained from linear regression and represent change in sialic acid concentrations per unit increase in BMI.

*BMI* body mass index.

^1^Adjusted for sub-study, race, gender, age at blood collection, and batch.

^2^Adjusted for covariates in Model 1 in addition to diet group.

## Discussion

Biological processes or alterations associated with cardiometabolic and other chronic diseases are still poorly understood. In this context, development of new biomarkers with recognized associations with such disorders is potentially helpful. Sias are known to accumulate in mammalian cells and tissues, and were proposed as biomarkers for some metabolic conditions^30,72–75^. If concentrations of Sias change with onset or progression of metabolic diseases, it is plausible that they are also impacted by dietary patterns which influence metabolic health.

In the current study we compared Sias among individuals following habitually vegetarian or non-vegetarian dietary patterns and found significantly higher levels of Neu5Ac in vegans and lacto-ovo-vegetarians relative to non-vegetarians. To our knowledge, this is the first study comparing Sias in adipose tissues from individuals in an epidemiological cohort specifically characterized by their dietary patterns. Hence, we compare our findings to other mostly in vivo studies reporting beneficial cardiometabolic effects of Neu5Ac supplementation. Such dietary supplementation has been found to have an intrinsic protective effect against atherosclerosis in mice^76^. Additionally, a reduction in coagulation-related cardiovascular events in hyperlipidemic conditions^26,77^, and prevention of clinical manifestations of cardiovascular disease have been documented^24,76^. Moreover, Neu5Ac dietary supplementation in experimental animals reduced the procoagulant, prothrombotic and antifibrinolytic effects of a high fat diet^24,76^.

Despite these promising findings, the usefulness of dietary supplementation with exogenous Sias in humans to reduce atherosclerosis, and consequently cardiovascular disease, remains questionable^29^. Furthermore, Sia levels in various human tissues have not been thoroughly investigated, and their physiological relevance is not well understood. Nevertheless, some trends have been identified. Obesity and systolic blood pressure in humans tend to be inversely associated with IgG sialylation^22^. This is consistent with in vivo studies demonstrating that dietary supplementation with N-acetyl-D-mannosamine (ManNAc), the first committed precursor of Neu5Ac, prevents obesity and systolic hypertension in mice, breaking the association between obesity and hypertension^78^. Additionally, increases in Sias due to dietary uptake appear to be associated with mechanisms that counter atherosclerosis, since Sias serve as substrates for re-sialylation of vascular endothelium. Thus, the presence of Sias on the vascular endothelium may alter these surfaces to reduce the risks of thrombosis^26^. However, the mechanisms for these effects are still largely unknown. Similarly, a positive impact on health by diet-related Sias, or by Sia supplementation, has not yet been established.

In contrast, there is evidence that total serum Sia is a risk factor for CVD, and correlates with CVD severity and mortality^79–81^. Similarly, increased plasma Sias are known to be associated with inflammation^82,83^. Since increased sialidase function is known to be associated with inflammation^84^, this could explain the higher circulatory Sias in these circumstances.

Given the pleiotropic roles of Sias, therefore, it is not unexpected for concentrations in serum or plasma to not correlate with those in adipose or other tissues. In this context, it appears that uptake of Sias by certain cell types may be associated with a dampened immune response^85,86^. Thus, while the physiologic relevance of higher adipose Sias in vegetarians is unclear, this may reflect a dampened immune or inflammatory response, relative to non-vegetarians.

KDN, similarly to Neu5Ac, was higher in vegans and lacto-ovo-vegetarians relative to non-vegetarians, though not reaching statistical significance, likely due to insufficient statistical power. However, similarly to Neu5Ac, KDN residues can be linked to almost all glycan structures. These linkage types include α2,3-, α2,4-, α2,6-, and α2,8-^87^. In this context, it might be expected that KDN and Neu5Ac follow a similar pattern of association with dietary groups.

The synthetic pathway for Sias is well described^9,88^. However, it is not clear why vegetarians would have higher Neu5Ac or KDN levels. While Sias are commonly present in dairy and animal products, they are not directly consumed from plant foods. Thus, it is not known to what degree (a) some raw precursors from natural sources in a vegan diet lead to increased incorporation of Sias into human tissues via de novo synthesis of Neu5Ac and KDN, or (b) some adverse impact of a non-vegetarian diet, such as increased inflammation, leads to reduction of Sias. Comparing vegans and non-vegetarians from the AHS-2 cohort, significant differences were found in monosaccharides, particularly metabolites in the fructose, mannose, galactose, and pentose subclasses, and other compounds that could impact precursors of Sias^89^. These vegans and other vegetarians, from the AHS-2 cohort, also have lower levels of pro-inflammatory compounds such as interleukin-6 and C-reactive protein relative to non-vegetarians^90^. Vegetarians also tend to have lower biomarkers related to meat or dairy consumption, lower saturated fatty acids and lipids known to be associated with cardiovascular disease and diabetes^89^. High consumption of red and processed meat has been associated with inflammation^91,92^. Hence, lower levels of Neu5Ac may imply a prolonged or higher baseline pro-inflammatory status.

Diet could also indirectly affect Sias through alterations in BMI or adiposity. In our study, KDN levels were lower with increasing BMI (Table 3), apparently independent of diet. While the relationship between BMI and Sias needs further elucidation, our findings are consistent with previous reports of lower Sias in obese individuals^78^. Compared to non-vegetarians, individuals following plant-based or vegetarian diets in AHS-2 were reported to have a lower risk of metabolic syndrome including lower BMI and waist circumference^93^, diabetes^94^, or cardiovascular disease mortality^54^. Therefore, the effects of diet on Sias could be attributable in part to adiposity. That is, a vegan diet promotes a lower BMI and anti-inflammatory environment, which may be associated with higher abundance of Sias in adipose tissue. The Sias in turn may help regulate or prevent the development of cardiovascular and other chronic diseases. From this perspective, a higher abundance of Neu5Ac might be expected in adipose tissue of vegans and lacto-ovo-vegetarians, considering the favorable cardiometabolic outcomes previously observed in vegetarians^49,95,96^. This is consistent with a role for Neu5Ac in the prevention of atherosclerosis^76^. Nonetheless, larger epidemiological or clinical studies are needed to specifically tease apart the contributions of diet and BMI to Sia levels.

In contrast to Neu5Ac and KDN, Neu5Gc was not detectable in human adipose tissue, although it was detected in animal adipose tissues from cows, lambs, and pigs. This is not surprising since Neu5Gc is known to be commonly expressed in mammals but not in humans^67,97–99^. Neu5Gc has been reported in some human tissues and fluids^16,27^, probably due to incorporation from food sources^11,100^. It has not been reported in human adipose tissue. This may be because Neu5Gc, if present, is below the current detection limits. It should be noted that even the non-vegetarian AHS-2 participants consume less red meat than the general population, or compared to participants of other studies such as the EPIC-Oxford^101^.

The relevance and potentially unique role of Sias in adipose tissue, in contrast to Sias in plasma or cancer cells, is noteworthy^32,33,102,103^. Although contributions from pathology-associated desialylation to human blood Sias remain speculative^84^, it is recognized that desialylation may lead to cellular dysfunction^104^. While Neu5Ac has been proposed as a potential factor counteracting atherosclerosis^76^, the source of Neu5Ac also needs to be considered. For example, sialidases may induce cleavage of Neu5Ac from the glycan chains of the LDL glycoprotein and glycolipids during the initial phase of atherosclerosis^29^. Following this, during CVD progression, while desialylation occurs in glycoconjugates, the serum sialic acid totals may increase^75,105^. Consequently, it could be hypothesized that in CVD, Sias may be released from adipose storage or other tissues as a compensatory response to reduce or counter inflammation. The development or discovery of biomarkers relevant to CVD will lead to more sensitive and effective screening approaches^106^.

Strengths of this study include the large number of habitual vegetarians and non-vegetarians in the Adventist Health Study-2 cohort, and the detailed diet and health history records for these participants. Additionally, the use of internal standards and other quality control procedures helped to ensure accurate and consistent measurement of Sias in human adipose samples. Furthermore, Sias were quantitatively measured using a direct and relatively straightforward method, without derivatization steps. The principal limitation of this study is its modest sample size. This likely reduced the statistical significance for the association between KDN and dietary pattern, and the contributing role of adiposity. Levels of free and conjugated Sias in the human adipose are not clear from the current study, as these were not measured separately, and consequently could not be compared. All Sia measurements were made using single time point biopsy samples, which may obscure seasonal or other temporal variations. Additionally, while adipose tissue samples are expected to largely include adipose cells, other cell types, including some vascular endothelial and fibroblast cells, were likely represented to some extent. Therefore, the source of Sias could not be fully ascertained. Moreover, Neu5Gc, which also has important implications for inflammatory or metabolic diseases, was below detection limits in human samples in the current study. Due to limitations of sample size, the analysis of associations of Sia concentrations with various physiologic parameters of interest have been limited to BMI. Finally, it would be useful to measure and compare adipose Sia concentrations with those in plasma or serum samples to further elucidate their biological roles. Such studies remain to be conducted.

## Conclusions

In the present study, we report successful detection of the Sias, Neu5Ac and KDN, in adipose tissue samples from individuals following habitual vegetarian and non-vegetarian diets. Neu5Ac was present at significantly higher concentrations in adipose tissue of vegans and lacto-ovo-vegetarians compared to non-vegetarians, while Neu5Gc was not detected in human adipose samples. KDN levels were modestly but significantly inversely associated with BMI. It is conceivable that dietary patterns regulate levels of Sias by influencing the accumulation of adipose tissue or by regulating inflammation. Such processes, in turn, may impact the onset or progression of chronic diseases.

This method for determination of Sias may be applied in future studies with human adipose tissues to quantify Sias and to elucidate their roles in human health or the development and progression of chronic diseases. In the longer term, assessing the impact of dietary patterns on Sia concentrations in human tissues will improve understanding of their associations with health and disease. Similarly, studies utilizing natural dietary components for de novo synthesis of Sias, may be helpful in the development of prevention strategies for cardiovascular and related diseases.

### Supplementary Information

Supplementary Information.

## Data Availability

Correspondence and requests for materials should be addressed to Fayth L. Miles.
