# Supplementary material for: Sialic acids Neu5Ac and KDN in adipose tissue samples from individuals following habitual vegetarian or non-vegetarian dietary patterns
Source: Sci Rep. 2023 Aug 3;13:12593. doi: 10.1038/s41598-023-38102-z (PMC10400564; doi:10.1038/s41598-023-38102-z)
Supplement: Supplementary file 1 — Supplementary Information. [file 41598_2023_38102_MOESM1_ESM.pdf]

Supplementary Materials

Sialic Acids Neu5Ac and KDN in Adipose Tissues from Individuals Following Habitual Vegetarian and Non-Vegetarian Dietary Patterns

Gerardo N. Guerrero-Flores[1,2], Fabio J. Pacheco[1,3], Danilo S. Boskovic[4], Sandaly O. S. Pacheco[1,3], Guangyu Zhang[4], Gary E. Fraser[5,6,7] and Fayth L. Miles[4,5,6]*

[1]Interdisciplinary Center for Research in Health and Behavioral Sciences, School of Medicine, Universidad Adventista del Plata, Libertador San Martín, Entre Ríos, 3103 Argentina
[2]Faculty of Medical Sciences, National University of Rosario (UNR), Rosario, 3100 Argentina
[3]Institute for Food Science and Nutrition, Universidad Adventista del Plata, Libertador San Martín, Entre Ríos, 3103 Argentina
[4]Division of Biochemistry, Department of Basic Sciences, School of Medicine, Loma Linda University, Loma Linda, CA, 92350 USA
[5]Center for Nutrition, Healthy Lifestyles and Disease Prevention, School of Public Health, Loma Linda University, Loma Linda, CA, 92350 USA
[6]Adventist Health Study, Loma Linda University, Loma Linda, CA 92350 USA
[7]Department of Medicine, School of Medicine, Loma Linda University, Loma Linda, CA 92350 USA

*Corresponding author: fmiles@llu.edu

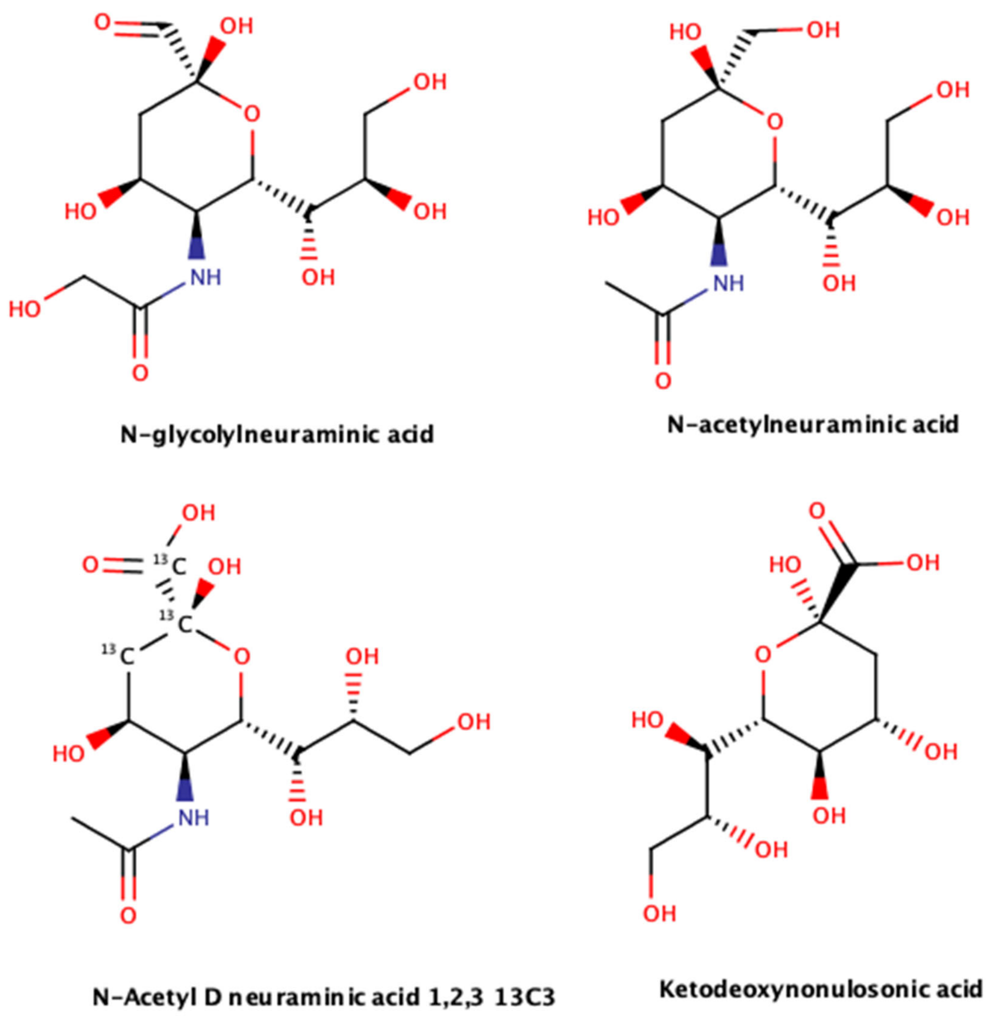

Supplementary Figure S1. Chemical structure of N-Glycolylneuraminic acid (Neu5Gc), N-Acetylneuraminic Acid (Neu5Ac), N-Acetyl-D-1,2,3-$^{13}C_3$ neuraminic acid (Neu5Ac-D-1,2,3-$^{13}C_3$) and Ketodeoxynonulosonic acid (KDN).

*Molecules were drawn and checked with the software MarvinSketch (version 22.13, academic package, ChemAxon).

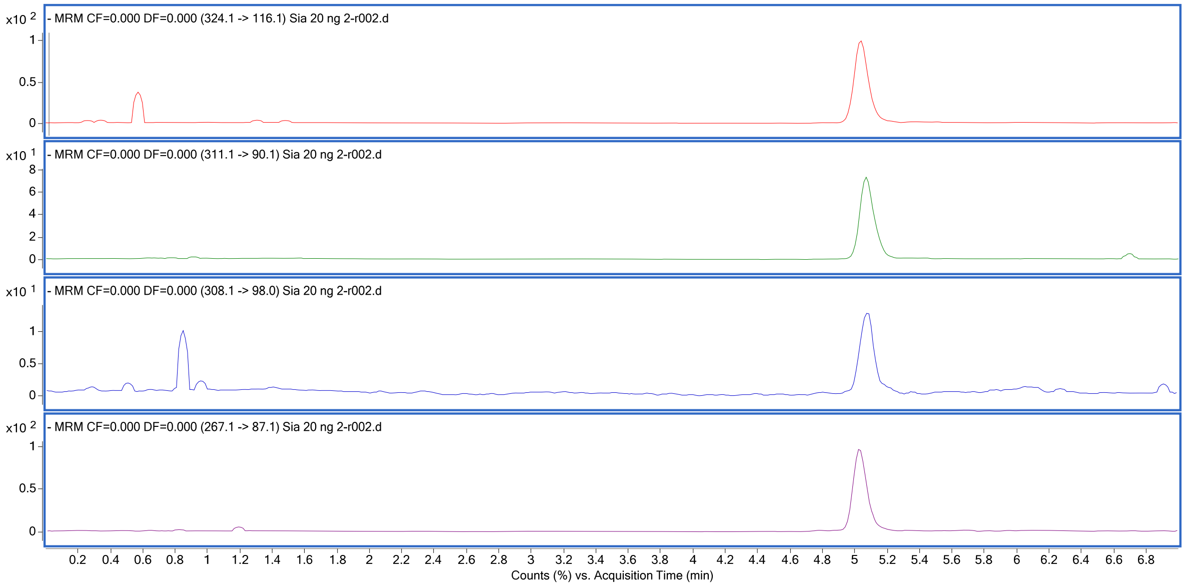

Supplementary Figure S2. HPLC chromatograms for quantification of Neu5Gc, Neu5Ac-D-1,2,3-$^{13}C_3$, Neu5Ac, and KDN content (20 ng/mL, each standard).

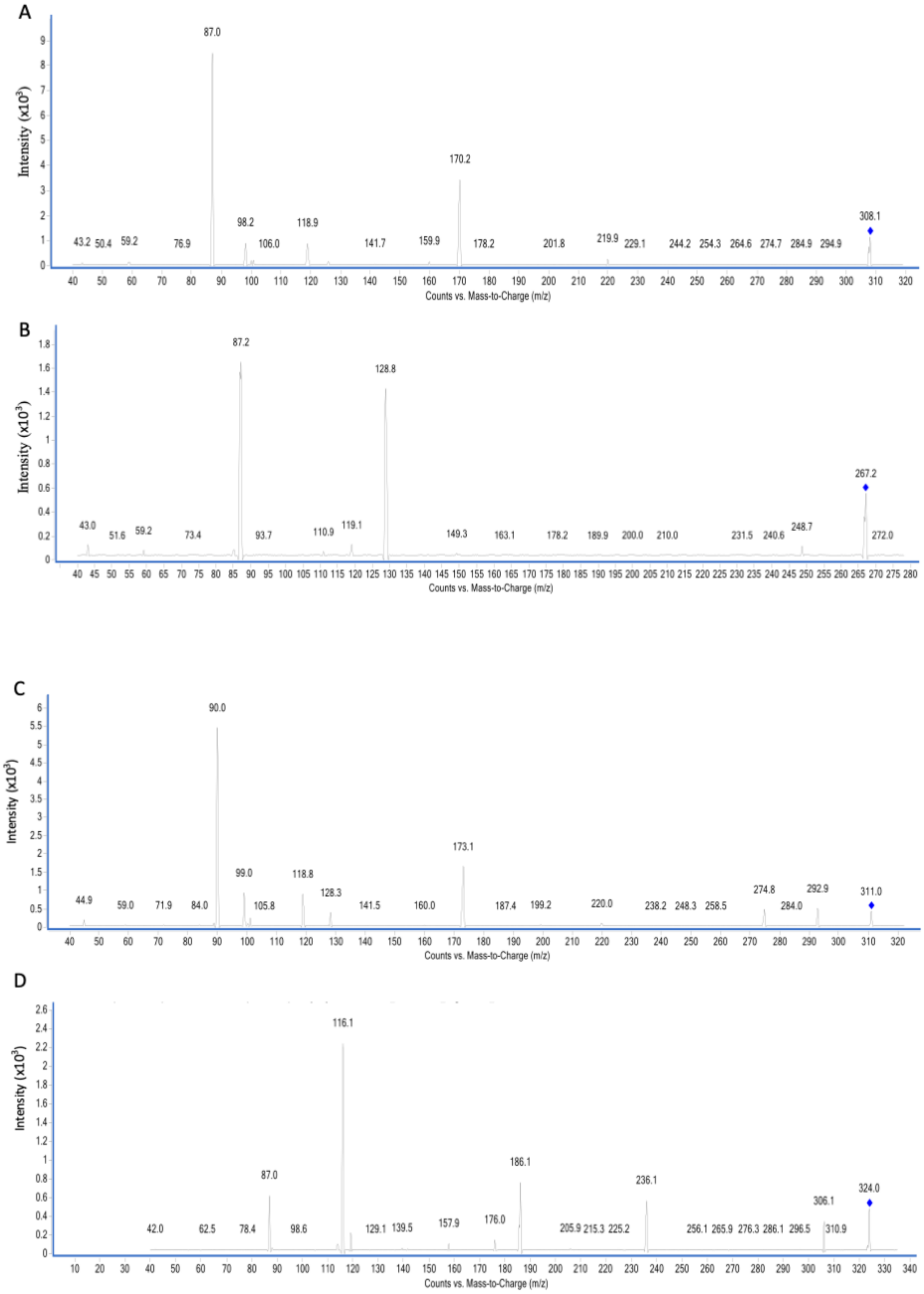

Supplementary Figure S3. Negative ionization electrospray mass spectra of the parent and daughter ion. A) Neu5Ac at m/z 87.0 with m/z 308.0 as the parent ion; B) KDN at m/z 87.2, with m/z 267.2 as the parent ion; C) Neu5Ac-D-1,2,3-$^{13}C_3$ at m/z 90.0 with m/z 311.0 as the parent ion; D) Neu5Gc at *m/z* 116.1, with m/z 324.0 as the parent ion.

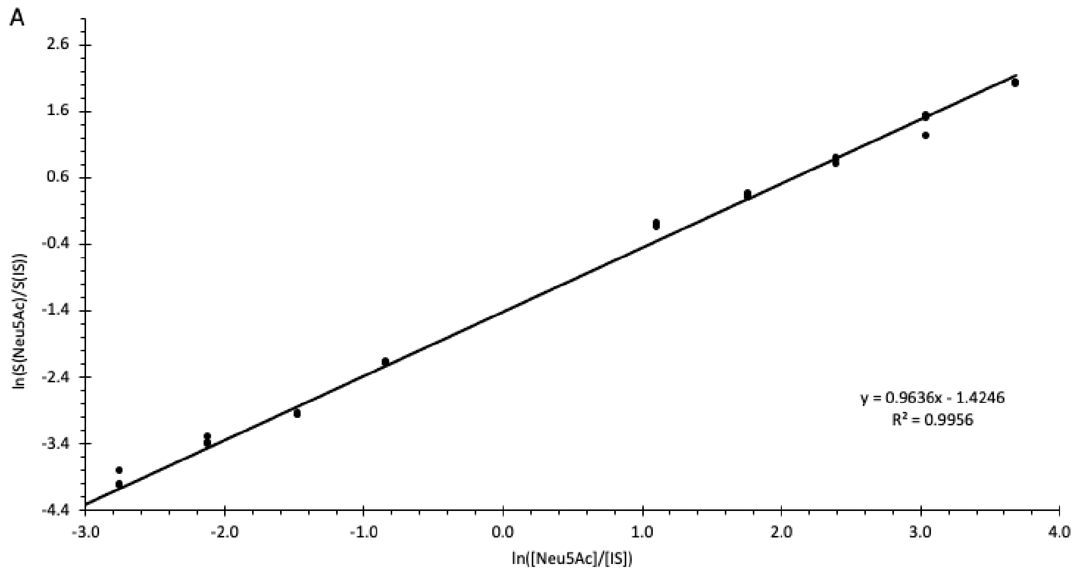
A
2.6
1.6
0.6
-0.4
-1.4
-2.4
-3.4
-4.4
ln(S(Neu5Ac)/S(IS))
-3.0
-2.0
-1.0
0.0
1.0
2.0
3.0
4.0
ln([Neu5Ac]/[IS])
y = 0.9636x - 1.4246
R² = 0.9956

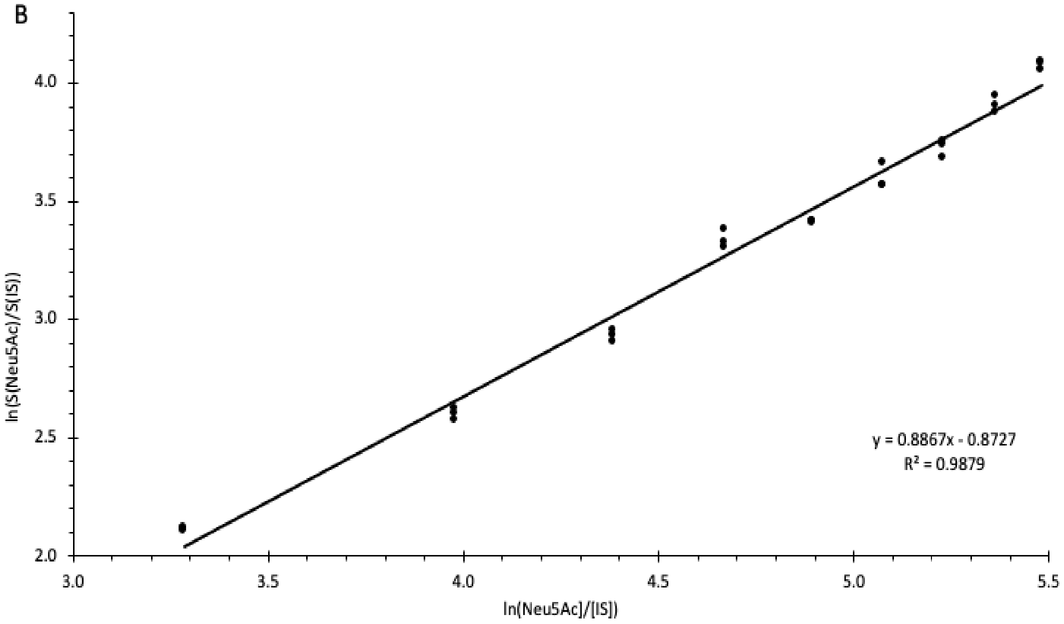
B
4.0
3.5
3.0
2.5
2.0
ln(S(Neu5Ac)/S(IS))
3.0
3.5
4.0
4.5
5.0
5.5
ln(Neu5Ac]/[IS])
y = 0.8867x - 0.8727
R² = 0.9879

C

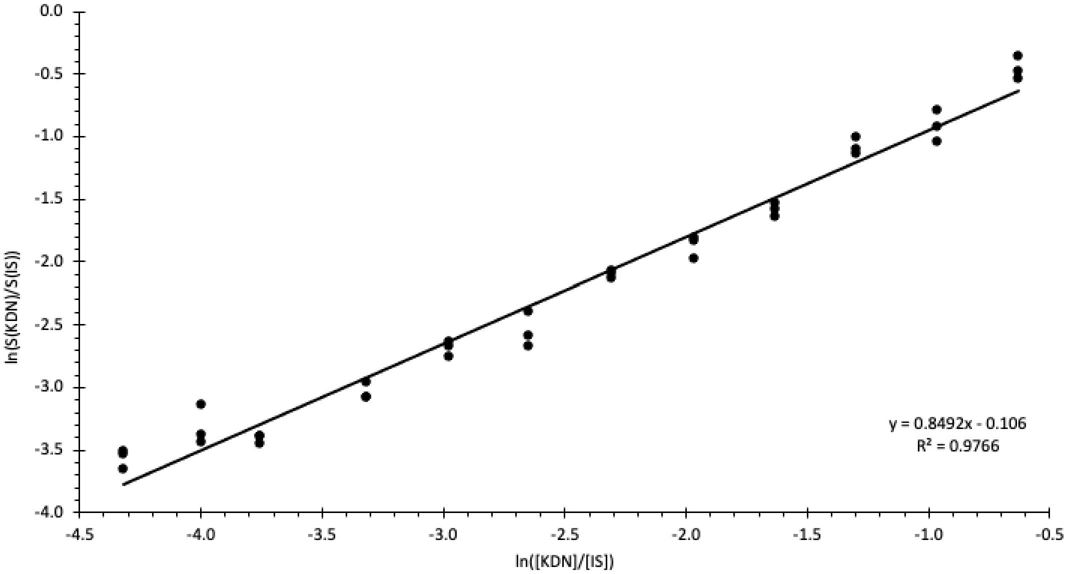
y = 0.8492x - 0.106
R² = 0.9766
ln(S(KDN)/S(IS))
ln([KDN]/[IS])
0.0
-0.5
-1.0
-1.5
-2.0
-2.5
-3.0
-3.5
-4.0
-4.5
-4.0
-3.5
-3.0
-2.5
-2.0
-1.5
-1.0
-0.5

D

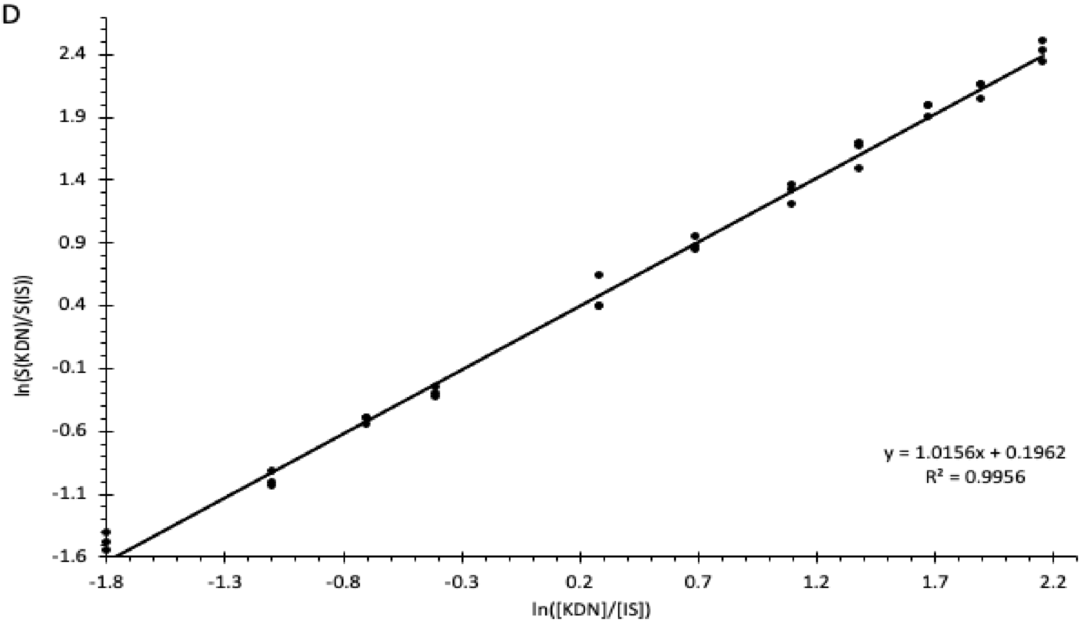
y = 1.0156x + 0.1962
R² = 0.9956
ln(S(KDN)/S(IS))
ln([KDN]/[IS])
2.4
1.9
1.4
0.9
0.4
-0.1
-0.6
-1.1
-1.6
-1.8
-1.3
-0.8
-0.3
0.2
0.7
1.2
1.7
2.2

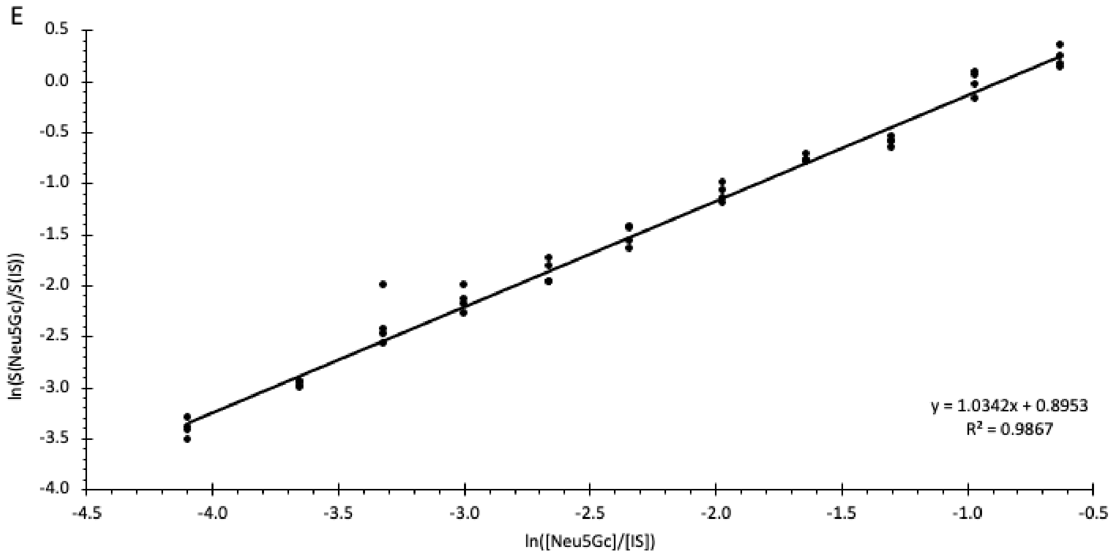

Supplementary Figure S4. The standard curves for the LC–MS/MS method for Sia quantitation, as a function of the ratios of the Neu5Ac (A-B), KDN (C-D), or Neu5Gc (E) concentrations to the concentration of the internal standard Neu5Ac-D-1,2,3-$^{13}C_3$ (IS).

The above standard lines were used to obtain respective inverse line equations as follows:

$$ln\left(\frac{[Neu5Ac]}{[IS]}\right) = 1.0378 * ln\left(\frac{S(Neu5Ac)}{S(IS)}\right) + 1.4784 \quad (A)$$

$$ln\left(\frac{[Neu5Ac]}{[IS]}\right) = 1.1277 * ln\left(\frac{S(Neu5Ac)}{S(IS)}\right) + 0.9842 \quad (B)$$

$$ln\left(\frac{[KDN]}{[IS]}\right) = 1.1776 * ln\left(\frac{S(KDN)}{S(IS)}\right) + 0.1248 \quad (C)$$

$$ln\left(\frac{[KDN]}{[IS]}\right) = 0.9847 * ln\left(\frac{S(KDN)}{S(IS)}\right) - 0.1932 \quad (D)$$

$$ln\left(\frac{[Neu5Gc]}{[IS]}\right) = 0.9669 * ln\left(\frac{S(Neu5Gc)}{S(IS)}\right) - 0.8657 \quad (E)$$

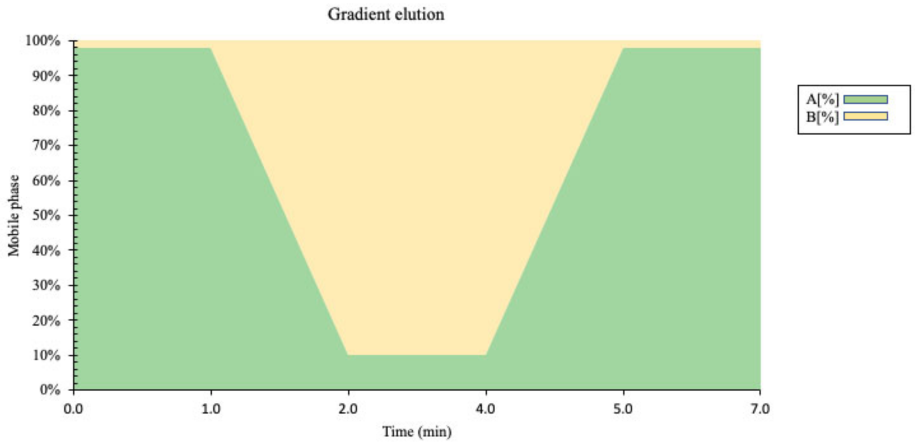

Supplementary Figure S5. HPLC gradient profile for the chromatographic separation of Sias. A= 10% ammonium formate in water; B= 10% ammonium formate in acetonitrile.

Supplementary Table S1. HPLC gradient profile for the chromatographic separation of Sias.

Time	Solvent A	Solvent B	Flow	Pressure
0.00 min	98.0 %	2.0 %	0.300 mL/min	400.00 bar
1.00 min	98.0 %	2.0 %	0.300 mL/min	400.00 bar
2.00 min	10.0 %	90.0 %	0.300 mL/min	400.00 bar
4.00 min	10.0 %	90.0 %	0.300 mL/min	400.00 bar
5.00 min	98.0 %	2.0 %	0.300 mL/min	400.00 bar
7.00 min	98.0 %	2.0 %	0.300 mL/min	400.00 bar

A = 10% ammonium formate in water; B = 10% ammonium formate in acetonitrile.

Supplementary Table S2. Parameters of the LC-MS/MS method for qualitative and quantitative determination of Neu5Gc, Neu5Ac, KDN and, Neu5Ac-D-1,2,3-$^{13}C_3$

Analyte	Retention time	Precursor ion (*m/z*)	Product ions (*m/z*)	Frag (V)	Collision energy (eV)	Quantitation transition
Neu5Gc	5.036	324.1	116.1	110	14	324.1 → 116.1
Neu5Ac	5.079	308.1	98	100	18	308.1 → 98
KDN	5.028	267.1	87.1	90	14	267.1 → 87.1
Neu5Ac-D-1,2,3-$^{13}C_3$	4.994	311.1	90.1	90	12	311.1 → 90.1

Supplementary Table S3. Contents of total sialic acids in animal adipose tissue (µg/mg).

Animal samples	**Neu5Ac**		**KDN**		**Neu5Gc**		**Total**	
n=3	mean	±SD	mean	±SD	mean	±SD	mean	±SD
Chicken	2.18	0.37	0.26	0.04	-	-	2.44	0.37
Pork	4.68	0.39	0.32	0.02	0.67	0.03	5.67	0.39
Lamb	2.78	0.33	0.28	0.06	0.54	0.04	3.60	0.34
Cow	3.13	0.56	0.11	0.10	1.73	0.16	4.97	0.59
